# High-grade extracellular vesicles preparation by combined size-exclusion and affinity chromatography

**DOI:** 10.1038/s41598-021-90022-y

**Published:** 2021-05-18

**Authors:** Cristina Bellotti, Kristina Lang, Nataliya Kuplennik, Alejandro Sosnik, Robert Steinfeld

**Affiliations:** 1 Department of Paediatric Neurology, University Children’s Hospital Zurich, University of Zurich, Zurich, Switzerland; 2 Department of Child and Adolescent Health, University Medical Center Gottingen, Göttingen, Germany; 3 Laboratory of Pharmaceutical Nanomaterials Science, Department of Materials Science and Engineering, Technion-Israel Institute of Technology, 3200003 Haifa, Israel

**Keywords:** Isolation, separation and purification, Nanobiotechnology, Microscopy, Multivesicular bodies

## Abstract

Extracellular vesicles (EVs) have recently gained growing interest for their diagnostic and therapeutic potential. Despite this, few protocols have been reported for the isolation of EVs with preserved biological function. Most EV purification methods include a precipitation step that results in aggregation of vesicles and most available techniques do not efficiently separate the various types of EVs such as exosomes and ectosomes, which are involved in distinct biological processes. For this reason, we developed a new two-step fast performance liquid chromatography (FPLC) protocol for purification of large numbers of EVs. The method comprises size exclusion chromatography followed by immobilized metal affinity chromatography, which is enabled by expression of poly-histidine tagged folate receptor α in the parental cells. Characterisation and comparison of the EVs obtained by this method to EVs purified by differential centrifugation, currently the most common method to isolate EVs, demonstrated higher purity and more selective enrichment of exosomes in EV preparations using our FPLC method, as assessed by comparison of marker proteins and density distribution. Our studies reveal new possibilities for the isolation of defined subpopulations of EVs with preserved biological function that can easily be upscaled for production of larger amounts of EVs.

## Introduction

Extracellular vesicles (EVs) refer to a group of naturally occurring small lipid bilayer particles that are derived from cells and cannot replicate^1,2^ including exosomes (EVs of endosome-origin^3–5^) and ectosomes (microparticles/microvesicles, plasma membrane-derived vesicles)^6,7^. Despite increasing interest in exosomes for their natural role in cell-to-cell communication and potential as diagnostic and therapeutic tools^8^, it remains difficult to selectively isolate them^9^. This is challenging due to shared characteristics with ectosomes, such as size, membrane architecture, density and many marker proteins. Proteins that are often considered “classical” exosome markers^10^ can be present at variable levels or can even be absent in some cases^11^ and have been found to be expressed in other subcellular compartments or by other types of EVs^12,13^.

There is currently no real gold standard for exosome isolation: although differential centrifugation (DC) is the most frequently used, many other methods have been proposed^14^. They are based on isolation by size, density or surface markers of exosomes, but each of them present some major disadvantage. Thus, the research for a technique that allows for recovery of a pure exosome preparation in an efficient, rapid, scalable and reproducible way continues to be of the utmost importance, especially if the objective is the use in a clinical setting^15^.

Corso et al.^16^ demonstrated that core bead chromatography using Capto Core 700 (CC700) is a suitable method to obtain EVs with a high yield and vesicular purity comparable to DC. CC700 beads have an inactive shell while their core is functionalized with both hydrophobic and positively charged ligands to guarantee multimodal binding of small contaminants. Particles smaller than 700 kDa are thus retained in the beads core, while EVs can be collected in the flowthrough. Since our interest lied mainly in exosomes, we aimed to optimize this method for exosome isolation.

**Figure 1:**
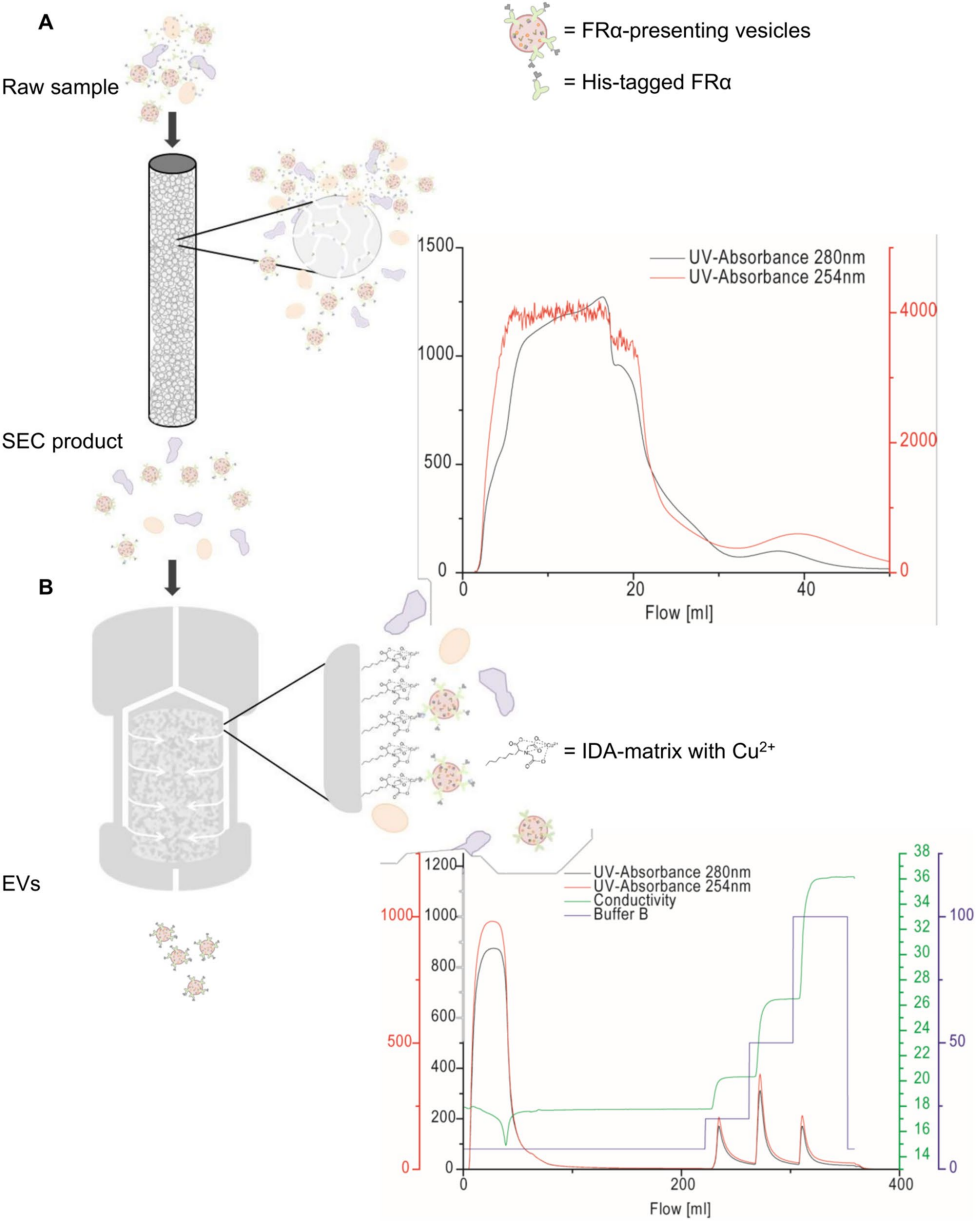
Scheme of two-step FPLC purification of his-tagged vesicles. (**A**) Samples with polyhistidine-tagged vesicles were applied to the SEC and small contaminating particles were retained in the hydrophobic and positively charged pores. The chromatogram resembled a flow-through rather than a gel filtration, as it is expected for this material, Capto Core 700. The sample volume in this example was about 20 ml. (**B**) Subsequently, the sample was applied to the copper-charged monolithic column. Polyhistidine-tagged particles bound to the Cu-atom and were then eluted with imidazole-containing Buffer B. As seen in the chromatogram, the major peak resulted from the second gradient step with 50% of buffer B.

Here, we present a two-step fast performance liquid chromatography (FPLC) protocol for exosome isolation consisting of size exclusion chromatography (SEC) using CC700 followed by immobilized metal affinity chromatography (IMAC). A scheme of the protocol is illustrated in Fig. 1. For selective isolation of exosomes in the later step of the purification, we needed to choose a membrane protein that is specifically enriched in endosome-derived EVs. For this purpose, we selected folate receptor α (FRα). FRα is expressed in epithelial cells^17^ and attaches to the cell membrane via a glycosylphosphatidylinositol (GPI) anchor^18^. FRα is naturally directed to the GPI-enriched early endosomal compartments (GEEC)^19^ and it was found to be secreted on exosomes by choroid plexus cells^20^. Considering its endosomal pathways and its natural occurrence on the exosomal surface, we considered FRα to be a reasonable candidate to distinguish between exosomes and other types of EVs. Therefore, we generated a HEK293 cell line expressing FRα with an N-terminal polyhistidine tag. The histidine tag enabled us to use IMAC to selectively isolate EVs carrying FRα on their surface and thus to obtain an exosome-enriched product with reduced ectosome contamination, which we refer to as an EV preparation.

We thoroughly characterized the obtained EV preparation to verify that it matches the minimal requirements defined by Lotvall et al.^2,21^ for EV samples: enrichment of EVs-specific markers and absence of non-EV proteins; appropriate size distribution as analyzed by nanoparticle tracking analysis (NTA); preservation of single vesicles shape and homogeneity of the preparation confirmed by electron microscopy.

As such, we confirmed that our purification method optimally preserved the biological characteristics of the EVs.

## Results

### Analysis of protein and particle yield by different purification methods

**Figure 2:**
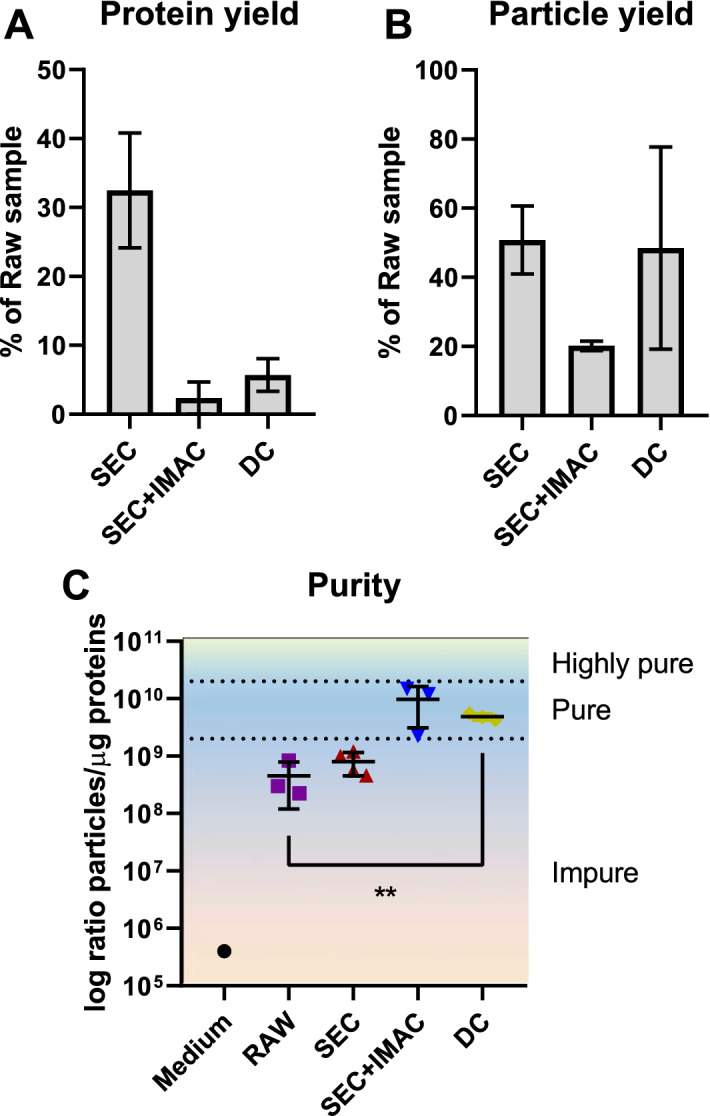
Yield and purity of EV preparation. Supernatant samples were purified either by the two-step FPLC method or by the conventional DC protocol. (**A**) Protein recovery from the intermediate SEC step (n = 3) and the final SEC + IMAC (n = 3) and DC (n = 4) products was estimated by a BCA assay and expressed as percentage of the protein content of the starting raw sample. (**B**) Particle recovery from the intermediate SEC step (n = 2) and the final SEC + IMAC (n = 2) and DC (n = 3) products was estimated with NTA and expressed as percentage of the particle content of the raw sample. (**C**) We considered the ratio number of particles/µg protein as a measure of purity of the EV preparation. Both the SEC + IMAC (n = 3) and the DC (n = 3) product fall in the range of what are considered pure EV preparations. Each symbol in the graph represents one sample. Data is represented as mean ± s.d. **= *p* < 0.01.

Amount of protein and number of particles in an EV preparation are commonly used parameters to quantify the output of an EV purification technique. Therefore, we measured the protein and particle concentrations of the starting material (raw sample, see Methods), of the SEC product and of SEC + IMAC and DC final products. The three elution peaks obtained with the FPLC method were measured separately and summed together to obtain the total yield of the method. Results are represented as percentage of the concentration of the raw sample in Fig. 2A,B. The data obtained indicate that the SEC step removed > 60% of the proteins from the raw sample. This step is particularly important to eliminate soluble FRα, which could interfere with vesicle isolation in the IMAC. Both protein and particle yield were higher after DC isolation than after the FPLC protocol, but the difference was not statistically significant.

### FPLC method allows for recovery of pure EV preparation

Purity of an EV preparation can be estimated by evaluation of different parameters, of which the most common is the particles per proteins ratio. In particular, preparations with less than 1.5 × 10^9^ particles/µg proteins are considered to be impure, preparations with 2 × 10^9^ to 2 × 10^10^ particles/µg proteins are considered pure, and preparations with > 3 × 10^10^ particles/µg proteins are considered as highly pure^22^. In our experiments, we determined this ratio for all steps of the FPLC method and for the final product of DC. Protein concentration of each sample was estimated with a bicinchoninic acid assay, while particle concentration was determined with NTA. Results are shown in a logarithmic scale in Fig. 2C. Both the SEC + IMAC and DC products fall in the same range of purity: the former has the highest amount of particles/µg proteins (9.69*10^9 ± 6.60*10^9 s.d. against 4.84*10^9 ± 6.43*10^8 s.d.), however, there is no significant difference between the two methods (Tamhane's T2 multiple comparisons test). We noticed a slight improvement in purity after the SEC step compared to the raw sample. This could be due to the fact that this chromatography step is supposed to remove small-size impurities that might fall under the detection limit of the NTA instrument (10 nm).

### Successful enrichment of EV markers and elimination of non-EV proteins in isolated EVs

In order to characterize the EV preparations, aliquots from each purification step of both the FPLC and DC methods were tested for presence of EV- and non-EV-markers by Western Blot (WB). Flotillin and Alix are commonly used EV markers, while TOM20 is a protein of the mitochondrial membrane. FRα expression has been reported to associate with EV-enriched proteins. 6-Pyruvoyltetrahydropterin Synthase (PTPS) is a protein that can be found in the cytoplasm or nucleus and was over-expressed in our cells as a control to check specificity of FRα enrichment.

**Figure 3:**
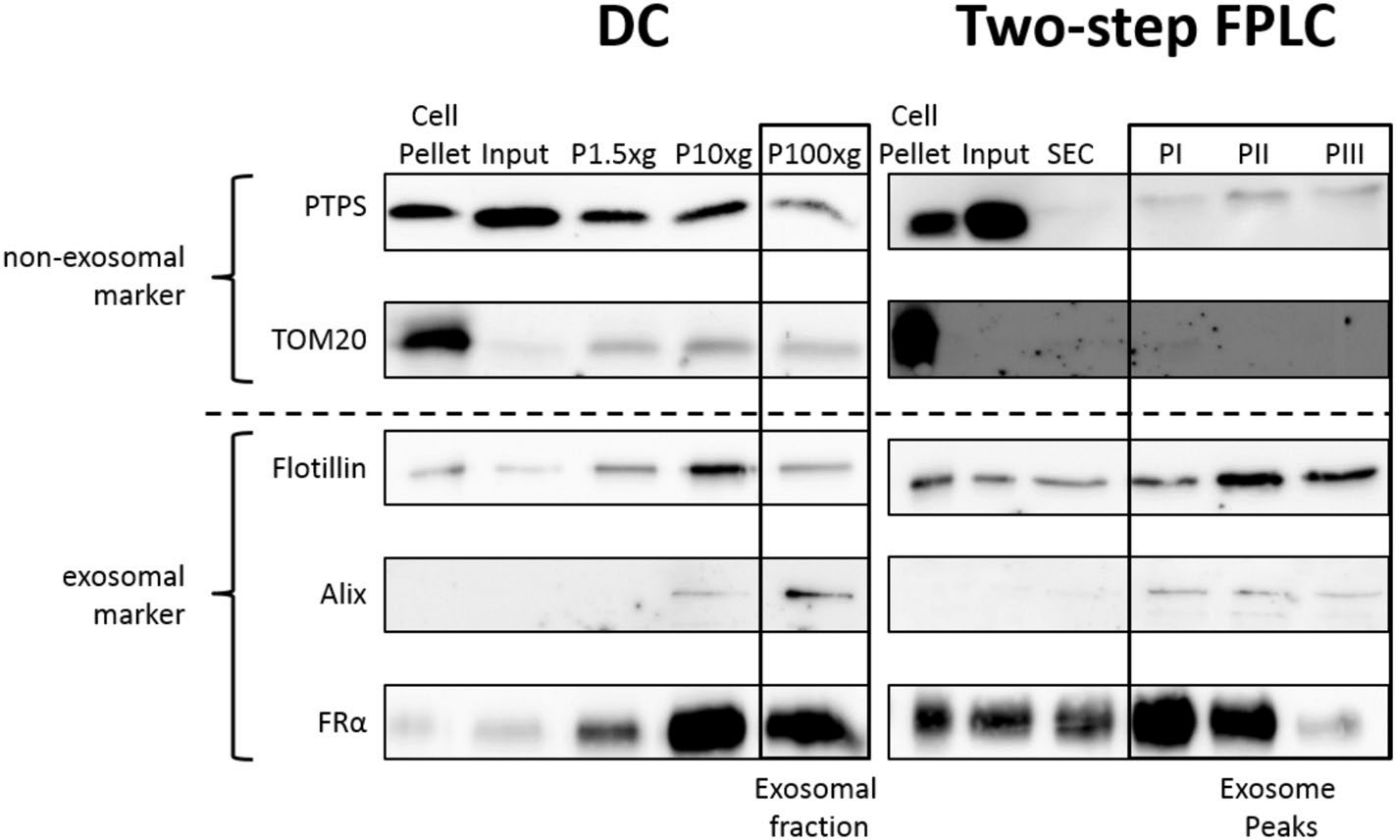
Characterization of EVs obtained by FPLC in comparison to EVs isolated by the DC protocol. EVs were prepared as described for the specific method. One sample from HEK293 cells expressing polyhistidine-tagged FRα was split into two halves to compare the expression of protein markers. For each step and method 1 µg total protein was loaded onto a gel. The blots were probed for EV-enriched proteins like FRα, flotillin and Alix. Additionally, the reduction of intra- and extracellular contaminations was investigated with the proteins TOM20 and the stably expressed PTPS. For this analysis we considered also the intermediate centrifugation steps of the DC protocol. Peak II (PII) of the two-step FPLC combining SEC and IMAC shows the strongest recovery of EV markers and reduction in EV-negative ones.

For this experiment, supernatant from polyhistidine-FRα overexpressing cells was purified either with the DC or the FPLC method. As demonstrated in Fig. 3, the DC product showed a strong enrichment of Alix and a less prominent enrichment of flotillin and FRα. Both TOM20 and PTPS were reduced in the final product compared to the previous steps, but they were still present in the sample. All EV markers were strongly enriched in the FPLC product (Fig. 3), with the second peak of elution of the IMAC (PII) appearing to be the best for enrichment of flotillin and FRα. Further, the presence of the non-EV markers was almost completely eliminated. In particular, PTPS appeared to be mostly removed from the sample after the SEC step.

### Size distribution of EV preparations

**Figure 4:**
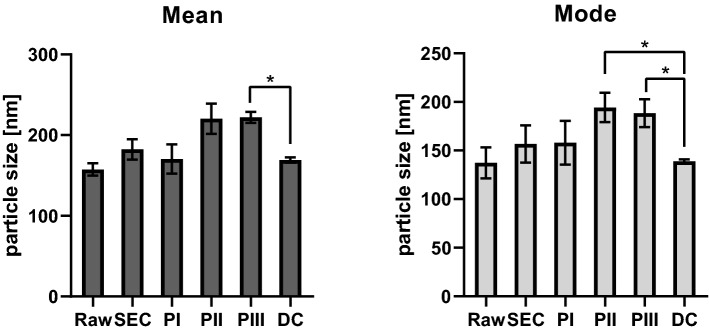
Analysis of mean and mode of vesicle size distribution in purified EV samples. Particle size was estimated with NTA, 3 technical replicates were registered per sample. Comparisons were made between the DC product (n = 3) and each peak of the FPLC final product (PI, PII, PIII, n = 3). * = 0.01 < *p* < 0.05. Data is represented as mean ± s.d.

Another important parameter for characterization of EV preparations is the size distribution of the particles. This was analyzed with NTA and results are shown in Fig. 4. For this analysis, we considered each elution peak from the IMAC column separately. Mean particle size of the EV samples obtained with DC was slightly smaller than that of samples obtained with our FPLC method, but the difference was statistically significant only in the case of PIII when applying the Sidak’s multiple comparison test of Nested 1 way ANOVA (*p* = 0.0490). EVs obtained with both protocols fell in the accepted range for EVs, even if the FPLC products had a mean particle size close to what is considered the maximum size of exosomes. We also analyzed the mode of the size distribution of EVs (Fig. 4), again comparing the DC product with each chromatography peak. In this case, the difference was significant with both PII and PIII (Sidak’s multiple comparison test of Nested 1 way ANOVA, *p* = 0.0103 and *p* = 0.0139). A mode considerably lower than the mean, as it appears to be for EVs obtained with DC, may indicate a substantial population of small particles in the sample.

### Density analysis of FPLC product suggests the presence of a single EV population

**Figure 5:**
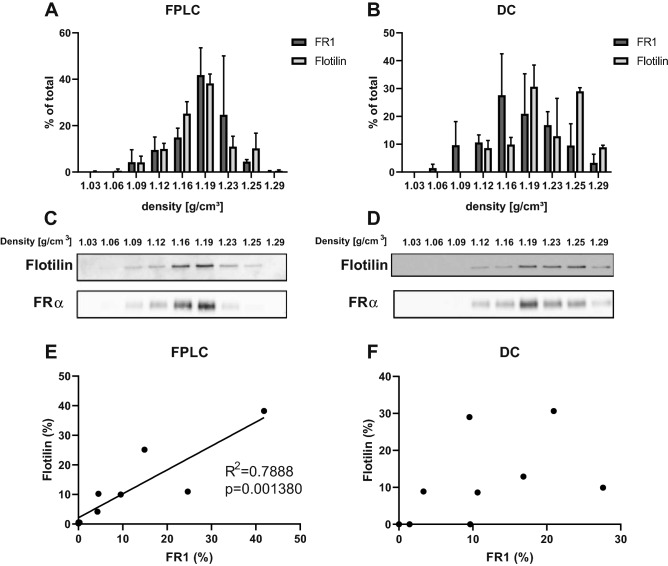
Analysis of EV markers distribution after centrifugation on a sucrose gradient. EVs isolated by FPLC (n = 2) or DC (n = 3 for FRα, n = 2 for flotillin) were centrifuged on a sucrose gradient, then samples from each density layer were analyzed by WB for the EV markers FRα and flotillin. (**A**,**B**) Pixel intensity was calculated with ImageJ. For each protein, all pixels of a sucrose gradient were summed and single bands were expressed as a percentage of the total pixel amount. Data is represented as mean ± s.d. (**C**) Example of WB of a FPLC-isolated and (**D**) DC-isolated EVs sample. (**E**,**F**) Correlation between FRα and Flotillin distribution across layers was significant for FPLC samples, but not DC samples.

For further characterization of the EV preparations, we tested their density by centrifugation on a sucrose gradient. After centrifugation, each density layer was tested for presence of EV markers flotillin and FRα. Data analysis (see Fig. 5 for details) demonstrated the highest amount of EV markers was found in layers corresponding to densities of 1.16–1.19 g/cm^3^ of the FPLC samples (Fig. 5A). This perfectly corresponds to the density that is expected from exosomes^23,24^. Results from the DC product (Fig. 5B) suggested instead the presence of two distinct populations of EVs, one FRα-positive with density 1.16–1.19 g/cm^3^ and one FRα-negative with density 1.25–1.29 g/cm^3^, a value that corresponds to ectosomes rather than exosomes. Figure 5C,D report examples of WB membranes from a single FPLC or DC sample. Further, we found that there is a significant correlation (*p* = 0.0014, R^2^ = 0.7888) between distribution of FRα and flotillin across the density layers for the FPLC product, but not for the samples obtained with DC (Fig. 5E,F).

### Presence of FRα on surface of isolated vesicles

**Figure 6:**
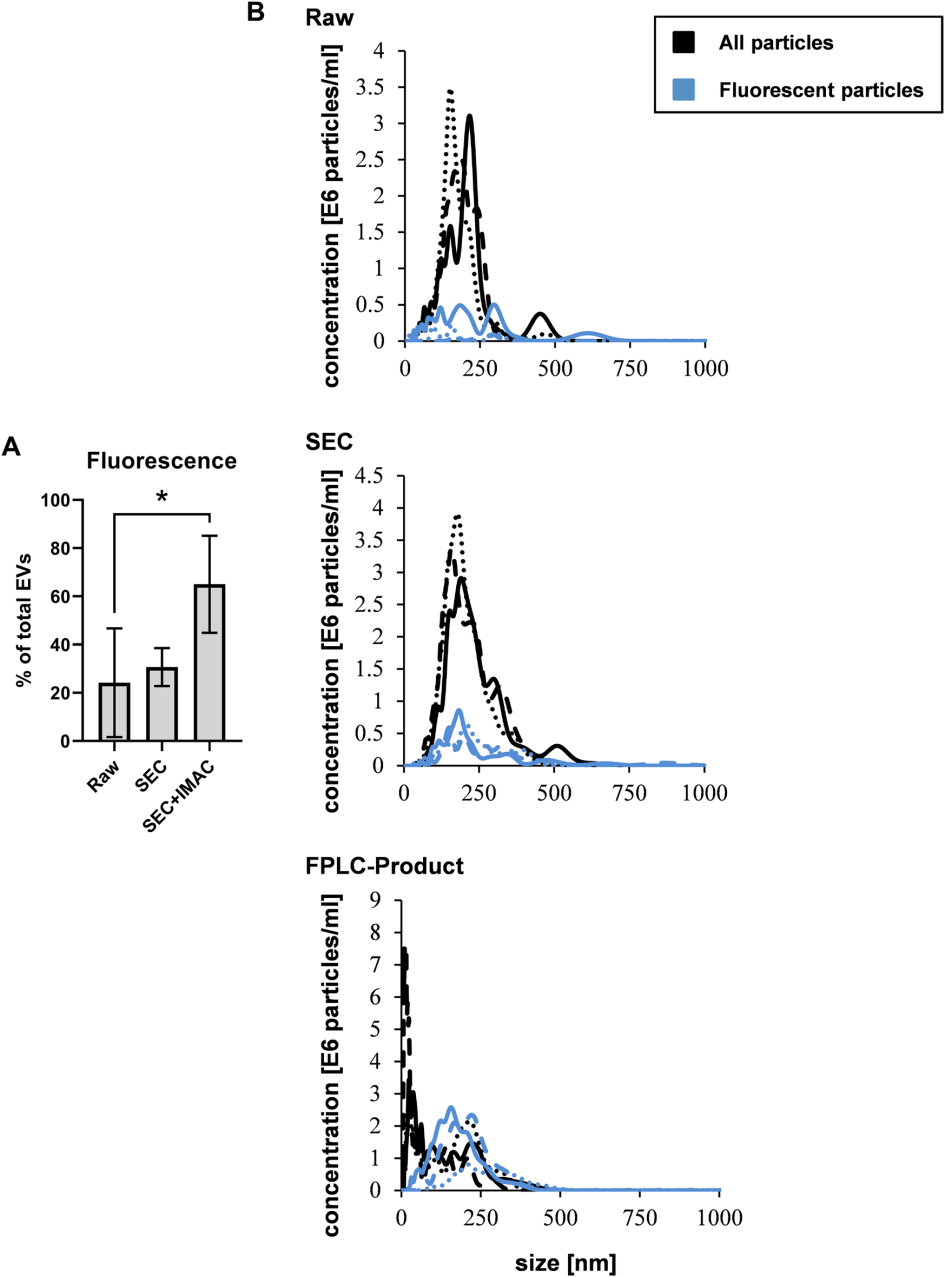
Analysis of fluorescent particles by indirect antibody labelling in NTA. Each preparation step (Raw n = 3, SEC n = 4, SEC + IMAC n = 3) was labelled with anti-FRα-antibody from mouse, washed and then labelled with a secondary antibody conjugated with anti-mouse IgG Alexa Fluor 532. The samples were not further processed but analysed by NTA. (**A**) Quantity of fluorescent particles is expressed as a percentage of the total number of particles. The amount of FRα-positive particles is increasing with each purification step. * = 0.01 < p < 0.05. Data is represented as mean ± s.d. (**B**) The size distribution of one analysis from three independent experiments is depicted as solid, dashed or dotted line. Measurement of all particles are represented in black, while fluorescent particles measurement are represented in blue.

To verify the specificity of our EV purification method, we investigated the presence of polyhistidine-tagged FRα on the surface of isolated EVs. Aliquots from either the raw sample, the SEC product or the second peak of the IMAC product were labeled with a primary anti-FRα antibody and then with a fluorescent secondary antibody. Total and fluorescent particle concentration of each sample, as well as the size distribution of both populations, was then estimated with NTA. In the bar graphs in Fig. 6A, the concentration of fluorescent particles is reported as a percentage of total particles in the sample. Fluorescent particles represented a significant percentage of the total particles within the raw sample. However, it is possible that part of these fluorescent signals originated from soluble FRα that was subsequently removed by SEC. The final FPLC product preferentially contained fluorescent particles (65% ± 20.12 s.d.) but a considerable number of FRα-negative particles remained. Considering the size distribution of the FPLC product, most of these unlabelled contaminants are smaller than EVs (< 30 nm) and thus may represent protein aggregates or membrane fragments (Fig. 6B). The one-way ANOVA test on the data confirmed a significant (*p* = 0.0421) difference between means. When comparing the final FPLC product with the previous purification stages, the difference with the raw sample was statistically significant (Sidak's multiple comparisons test, *p* = 0.0425).

### EM imaging of single vesicles confirms high quality of EV preparation

**Figure 7:**
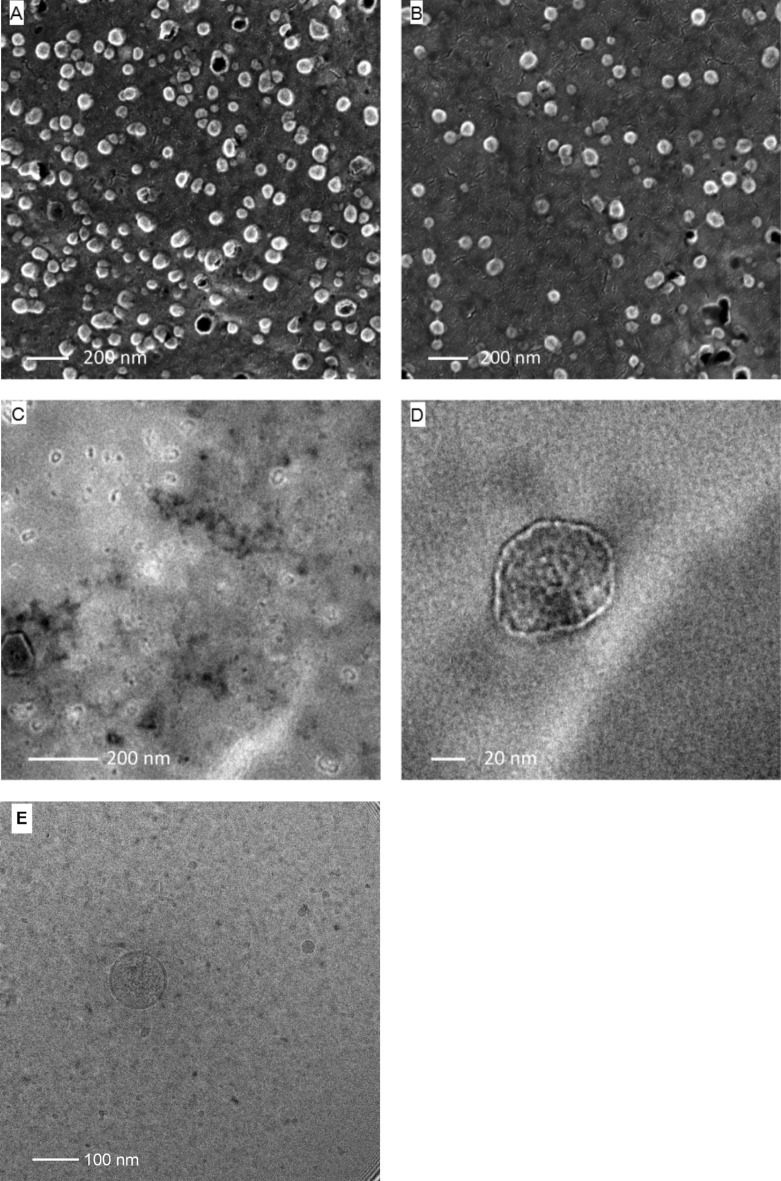
Electron microscopic examination of vesicles purified by SEC and IMAC. Samples were prepared by the two-step FPLC method as described in methods. (**A**,**B**) FRα-expressing exosomes from HEK293 cells imaged by scanning electron microscopy. The vesicles are of exosome-like size 40–200 nm and cup shaped. (**C**) using transmission electron microscopy it was possible to verify homogenous size distribution of the vesicles as well as (**D**) the presence of a double membrane. (**E**) cryo-TEM imaging of the exosomes confirms the spherical shape and the size of approximately 120 nm as well as the lipid bilayer membrane.

Characterization of single vesicles was carried out by electron microscopy (EM) imaging. Scanning electron microscopy (SEM) images of EV samples obtained by SEC + IMAC purification shows particles with a cup-shaped morphology typical of exosomes. Black holes visible in Fig. 7A,B are due to larger vesicles that were sucked by the vacuum. The preparation appears uniform, with almost no debris contamination; ectosome-like particles were not detected.

Transmission electron microscopy (TEM) allows for higher resolution-imaging, but it has the disadvantage of fast sample degradation. With this technique it was possible to confirm the homogenous size distribution of the sample and even visualize the double-layered membrane of single vesicles (Fig. 7C,D). Using cryo-TEM we could demonstrate the spherical shape as well as the size of the exosomes matching with the data obtained by the NTA (Fig. 7E).

## Discussion

The major challenge when working with EVs is represented by their purification and by the enrichment of specific types of EVs such as exosomes in such a way as to preserve their biological function.

DC is quite easy to perform and relatively inexpensive, but subjects the vesicles to high mechanical stress that can alter their morphology^25^; in addition, vesicles tend to form aggregates when pelleted that cannot be completely resuspended^26^. Moreover, samples often contain contaminants that can hinder the exosome’s biological activity^27^, and EV recovery is affected by the viscosity of the medium^28^. Other methods can solve some of these problems but come along with other disadvantages. Density gradient ultracentrifugation has gained popularity because it can reduce contaminants and yield higher purity EVs^27,29^ but it does not reduce mechanical stress. Further, sample volume is rather limited and this method cannot easily be scaled up. Another common strategy for exosome isolation is polymer-induced precipitation, for example using polyethylene-glycol (PEG) to precipitate exosomes. PEG causes exosome solubility to change, so that they can be precipitated with low-speed centrifugation. This method is easy to perform, it does not require an ultracentrifuge, it reduces mechanical stress and it has a high yield^30,31^. On the other hand, PEG and similar polymers can precipitate also protein aggregates and other contaminants that have biophysical properties comparable to exosomes^29,31^. In addition, this protocol requires complicated clean-up steps and samples may still be contaminated by the used polymer, which in turn can have cytotoxic effects^31,32^. In general, most of these methods are optimized for small sample volumes and are not suitable for large-scale purification of any type of EVs and industrial production of EVs (for example for clinical application of EVs as drug vehicles).

Other techniques rely exclusively on size to isolate exosomes. Between these, ultrafiltration has gained popularity for its easy and fast approach to exosome purification, which can provide yields similar to those obtained with ultracentrifugation^33^. The drawbacks of this method are the risk of vesicle clogging the filter and of contamination by nanoparticles in the same size range of exosomes^33^. Further, transmembrane pressure can affect exosome function. SEC approaches are also relatively easy and fast to implement, and have the advantage of preserving vesicle integrity and function compared to other methods^32^. However, samples obtained with SEC are low in purity, so this method is usually applied in combination with another technique, such as ultrafiltration^34^ or ultracentrifugation^35^.

Immunoaffinity-based techniques rely on the reaction between exosome surface proteins and an immobilized antibody, e.g. conjugated with magnetic beads. While this allows for the recovery of a highly pure exosome sample compared to other methods^36^, the reagents needed are usually expensive and the sample volume that can be processed is very limited. In addition, the lack of a universal yet selective exosome marker implies that only subpopulations of exosomes expressing a specific protein can be captured. This can be exploited for diagnostic purposes allowing the specific isolation of exosomes that express defined targets of interest or are produced by a determined cell line^37^.

It is also worth to mention that methods using microfluidic systems have been demonstrated to have great potential for fast and pure exosome isolation but are again limited to small sample volumes^38^.

We have developed a FPLC purification protocol for the isolation of EVs with defined protein marker characteristics that preserves the natural EV morphology, avoid formation of aggregates, and can be readily scaled up. We compared this FPLC protocol with the DC procedure as a control to substantiate the superior efficiency and quality of this new method.

When we quantified protein and particle recovery of the two methods, we found that the FPLC protocol had a lower albeit not statistically significant yield than DC. This might be explained by the presence of non-EV particles after the DC isolation and/or the selective enrichment of subpopulations of EVs by the FPLC method. The fact that EV markers only correlated with each other in the case of the FPLC method (Fig. 5) supports this hypothesis. Further, the protein release by cells, the protein content per particle and the relative contribution of various types of EVs that are secreted by cells are not considered in the calculation of protein and particle yield. The poor reproducibility of exosome isolation, particularly using ultracentrifugation, has already been object of other studies^39^.

The purity of the EV preparation, expressed as the ratio of particles/proteins, was not significantly different between the two methods. The same was true for size distribution of the preparations: the mean size for both methods fell into what is considered the exosome size range, even if the FPLC product tended to be more towards the high end of it. Both methods resulted in enrichment of EV markers, while FPLC seemed to be even more efficient than DC in reducing non-EV markers. In fact, considering the presence of non-EV markers the DC product still contained almost 20% of the concentration of PTPS of the raw sample, while in each elution peak of the FPLC product PTPS concentration was < 7% of that of the input; TOM20 appeared to be almost 3.5 folds more concentrated in the DC product than in the raw sample, but it was barely detectable only in PI for the FPLC product (see Fig. 3). In addition, when density of particles was considered, the FPLC product showed a more homogenous population than EVs obtained by DC. This suggests a higher efficiency of FPLC in reducing contaminants and indicates a major advantage for our method. Further, we have to consider that the IMAC step will result in the exclusive isolation of particles that express FRα. The current hypothesis is that several pathways of EV production exist^40^ and consequently different subpopulations of EVs^41^. It is possible that FRα is present only on the surface of specific subpopulations of EVs. In addition, the presence of several EV populations explains the lower homogeneity of the DC product. Altogether these results underline the importance of characterizing the protein profile of the product of a EVs purification protocol and not relying only on parameters like number of particles per µg of protein to estimate its purity.

When we assessed the percentage of FPLC-isolated EVs that were immunolabelled for FRα, we could still detect a population of FRα-negative particles. The unlabeled particles were mostly very small (< 30 nm) and represented a population that was not present in previous stages of purification (Fig. 6B). These ultrasmall particles may comprise non-biological material derived from buffer solutions or column matrices. The fact that these ultrasmall particles were mainly present in the final purification step may relate to specific contaminations during this procedure. In particular, the imidazole present in the buffer used to elute the EVs from the IMAC column may have interfered with the ultrafiltration device used to wash out the primary antibody solution from the samples. This would explain why these contaminants are only present in the final FPLC product and why this consistent population of small particle was not detected when size distribution of the samples was analyzed (see “Size distribution of EV preparations” in the Results section and Fig. 4B).

Finally, we characterized FPLC-purified EVs by electron microscopy to confirm the presence of particles with a lipid bilayer, the expected shape of exosomes and the homogeneity of the sample preparation. Further experiments are required to describe the functionality of these EVs and to validate their biological effectiveness after uptake by target cells.

In conclusion, we established a novel two-step FPLC protocol for EV isolation that matches the minimal requirements for EV sample preparation. We additionally demonstrated that this method enables the recovery of EVs with similar characteristics when compared with DC, which is currently the most used method for EV isolation. Comparison of the marker proteins and density distribution of EVs indicated that our method was even more efficient than DC in enriching exosome type of EVs and thus represents a promising alternative to this widely used technique. In particular, considering the large sample volume that can be processed at once via FPLC and the high potential for automation of the protocol, our method can be easily scaled up and thus may be utilized in industrial settings.

## Materials and methods

### Generation of FRα overexpressing cell line

Human embryonic kidney HEK293 cells were purchased from ATCC and cultured in Dulbecco’s Modified Eagle’s Medium (DMEM) with 5% Fetal Calf Serum (FCS) until transfection. A pEFTT plasmid with one copy of N-terminally polyhistidine-tagged FRα and one copy of the *pts* gene for control was obtained with restriction cloning. Cells were transfected with the plasmid via lipofection. In parallel, a transfection with the empty plasmid (not containing polyhistidine-FRα) was carried out to generate a control cell line. Selection was performed adding 1 μg/ml Puromycin (InvivoGen) and 50 μg/ml Geneticin (InvivoGen) to the media. Single clones were isolated and WB was performed to determine FRα expression. Highest expressing clones were chosen and slowly adapted to serum-free medium 293 SFM II (Thermo Fisher Scientific) supplemented with 1% Glutamax (Thermo Fisher Scientific) and 2 μg/ml Ca-folinate.

### Cell culture and sample preparation

To scale up EV production, cells were cultured in a miniPERM bioreactor (Sarstedt) with a production module of 35 ml according to the manufacturer’s directions. The bioreactor was kept on a rotating platform (Universal Turning Device, Sarstedt) in an incubator at 37 °C and 5% CO2.

HEK293 cells overexpressing FRα or transfected with the empty plasmid were cultured in T175 flasks until they reached high density. Then, cells from several flasks were combined and cultured in the production module. The nutrient module was filled with 350 ml of DMEM high glucose with 5% FCS.

The cell suspension in the production module was collected after 4 days. Supernatant and cells were separated by low-speed centrifugation and half of the cells were returned to the production module. The remaining cells were replaced by new cells from T175 flasks. Supernatant samples were then centrifuged at 4500 rpm for 30 min at 4 °C and passed through a 0.4 μm filter. This is referred to as “raw sample”.

### Fast performance liquid chromatography

All chromatography protocols were performed using an ÄKTA Purifier 10 system (GE Healthcare).

The first purification step consisted in a SEC. Up to 130 ml of raw sample were applied to a HiScale 16/20 column (GE Healthcare) packed with 20 ml of Capto Core 700 (GE Healthcare). PBS (pH 7.4) was used to finalize sample application. For the chemical characteristic of the matrix, small (< 700 kDa) contaminants such as proteins are retained in the beads and are eluted from the column only during the column wash with NaOH and 30% isopropanol, while bigger objects bypass the beads and are eluted immediately. Thus, the chromatogram resembles a constant elution during sample application, without distinct peaks (Fig. 1A). For this reason, some scientists refer to this methodology as bind-elute size exclusion chromatography^16^. The whole first elution fraction (containing particles > 700 kDa) was collected and applied to the IMAC column.

An IMAC constituted the second purification step. A CIMmultus IDA monolithic column, 6 μm pores (BIA Separations), was charged by washing with a CuSO_4_ solution according to the manufacturer's instructions. For the IMAC protocol, two buffers were used: Buffer A (140 mM NaCl, 20 mM phosphate, pH 7.4) and Buffer B (Buffer A with 500 mM imidazole, pH 7.4). Imidazole is necessary in the elution steps to displace bound histidine from the column. 8% volume of Buffer B was added to the SEC product prior to application to the column to reduce non-specific binding. The column was equilibrated by washing with Buffer A plus 8% Buffer B for 20 column volumes (CV). Following sample injection, the column was again washed with Buffer A plus 8% Buffer B, then three elution steps of 5 CV each were performed, increasing Buffer B concentration respectively to 20, 50 and 100%. Elution peaks were collected in 2 ml fractions. All fractions constituting a single peak were then recombined and elution peaks were analyzed separately. For density centrifugation, fluorescent labelling and electron microscopy, only PII was used.

Each column was regenerated according to the manufacturer instruction after every purification.

### Differential centrifugation

Supernatant samples from HEK293 cells with and without polyhistidine-FRα were purified with a DC protocol. Samples were subjected first to a slow centrifugation (1500 g for 15 min at 4 °C) to remove big debris, then to a second one (10,000 g for 45 min at 4 °C) to remove smaller contaminants. EVs were finally pelleted with an ultracentrifugation step (100,000 g for 2.5 h at 4 °C).

### BCA protein estimation

To assess protein content of the samples, the Pierce BCA Protein Assay Kit (Thermo Fisher Scientific) was used. For this 25 μl of each sample and 200 μl of reagents mix were pipetted in a 96-well plate. Reactions were incubated at 37 °C for 30 min and the linear absorbance at 562 nm was read with a photometer. Imidazole has a non-negligible absorbance at 560 nm and can thus create artefacts in the assay. For this reason, samples which contained imidazole from the elution buffer used in the IMAC protocol were washed in an ultrafiltration unit prior to measurement.

### Western blot

Defined amounts of protein per sample was diluted in 4 × SDS-PAGE sample buffer, denatured for 10 min at 95 °C, and loaded on a 10% or 15% acrylamide gel. Transfer to a nitrocellulose membrane (Amersham) was performed in a semi-dry blotting instrument, applying 1 mA/cm^2^ of membrane surface for 90 min. The membrane was incubated for 1 h in blocking solution (PBS-T with 5% w/v powder milk). All antibodies were also diluted in blocking solution (anti-AIP/Alix, 1:500 [ABC40, Sigma- Aldrich], anti-Flotillin2, 1:400 [610383, BD Biosciences], anti-FRα, 1:2000 [NCL-L-FRalpha, Leica Biosystems], anti-PTPS, 1:2000 [PA5-22121, Thermo Fisher Scientific], anti-TOM20, 1:1000 [sc-11415, Santa Cruz Biotechnology], anti-mouse IgG HRP-conjugated, 1:10,000 [Jackson ImmunoResearch Laboratories], anti-rabbit IgG HRP-conjugated, 1:5000 [Jackson ImmunoResearch Laboratories]). Membranes were incubated in primary antibody overnight at 4 °C, followed by 3 5-min washes in PBS-T and 1 h incubation in secondary antibody solution RT. Lumi-Light^PLUS^ Western Blotting Substrate (Sigma-Aldrich) was used to visualize the peroxidase-conjugated secondary antibody in the Luminescent image reader LAS-400 mini (Fujifilm). WB images were analyzed using ImageJ (National Institutes of Health).

### Analysis of density by centrifugation on a sucrose gradient

A discontinuous gradient of sucrose in HEPES buffer (Carl Roth GmbH + Co. KG) was prepared starting from a 2.5 M sucrose stock solution. Sucrose concentration in each fraction of the gradient ranged from 2.25 to 0.25 mM (corresponding to 1.29–1.03 g/cm^3^ density) in steps of 0.25 mM. For this 700 μg of proteins from the sample (EVs exclusively from polyhistidine-FRα overexpressing cells purified either by DC or FPLC) were added to the 0.25 mM sucrose fraction. 2.7 ml of each fraction were carefully layered in a tube and centrifuged at 26,000 rpm (corresponding to 100,000 g) for 16 h at 4 °C using a SW28 rotor (Laborgeräte Beranek GmbH). Fractions were then collected separately and an equal volume of each fraction was analyzed by WB.

### Nanoparticle tracking analysis and fluorescent labelling of FRα

EV samples were analyzed using a NanoSight LM10-HS (Malvern Panalytical) equipped with a high sensitivity CMOS camera, a LM14 viewing unit with 532 nm laser and a fluorescence filter. To obtain a particle concentration compatible with the instrument (2 × 10^8^–10^9^ particle/ml), raw samples were usually diluted 1:10,000, SEC product 1:1000, the final FPLC product 1:10, DC product 1:10,000 in PBS. 0.5 ml of diluted sample were applied to the sample chamber and recorded in triplicates for 30 s. Sample parameters were calculated by NanoSight NTA 2.3 software. Particle concentration, mean and mode particle size values as well as the size distribution graph were extracted for further analysis.

For fluorescent labeling of EVs, particle concentration was adapted to 10^10^ particles/ml. Anti-FRα antibody (1:400, ALX-804-439, Enzo Life Sciences) was added to 0.5 ml sample and incubated for 1 h at 4 °C with mild agitation. An Amicon Ultra ultrafiltration unit 50 K (Sigma-Aldrich) was used to remove excess antibody by washing with 5 × 500 μl PBS and centrifuging at 3500 g. Fluorescently labeled secondary antibody (anti-mouse IgG Alexa Fluor 532-conjugated, 1:800 [A11002, Life Technologies]) was added to the sample and incubated for 30 min at 4 °C. Samples were then applied to the NTA instrument without further washing. To avoid photobleaching, a constant flow was applied to the sample during recording. Flow rate was set according to manufacturer’s directions.

### Electron microscopy

For Scanning Electron Microscopy (SEM), 2 μl of EV suspension were dropped on a carbon tape, dried and covered with Au/Pd-coating. For Transmission Electron Microscopy (TEM), samples were dropped onto a Formvar/carbon FCF300-Cu grid (Agar Scientific Ltd), dried at RT and imaged with a Tecnai G2 T20 S-Twin TEM (FEI Company). Cryo-TEM imaging was performed in a FEI T12 G2 electron microscope (FEI Company) operated at 120 kV as described before^42^.

### Statistical analysis

Statistical analysis was performed using GraphPad Prism 8 (GraphPad Software). The threshold to accept statistical significance was set at alpha level 0.05 for all p-values.

## Supplementary Information

Supplementary Information.

## Data Availability

The data generated during the current study are available from the corresponding author upon reasonable request.

## References

[1] Yanez-Mo M (2015). Biological properties of extracellular vesicles and their physiological functions. J. Extracell. Vesicles.

[2] Thery C (2018). Minimal information for studies of extracellular vesicles 2018 (MISEV2018): a position statement of the International Society for Extracellular Vesicles and update of the MISEV2014 guidelines. J. Extracell Vesicles.

[3] Harding C, Heuser J, Stahl P (1983). Receptor-mediated endocytosis of transferrin and recycling of the transferrin receptor in rat reticulocytes. J. Cell Biol..

[4] Pan BT, Teng K, Wu C, Adam M, Johnstone RM (1985). Electron microscopic evidence for externalization of the transferrin receptor in vesicular form in sheep reticulocytes. J. Cell Biol..

[5] Johnstone RM, Adam M, Hammond JR, Orr L, Turbide C (1987). Vesicle formation during reticulocyte maturation: association of plasma membrane activities with released vesicles (exosomes). J. Biol. Chem..

[6] Raposo G, Stoorvogel W (2013). Extracellular vesicles: exosomes, microvesicles, and friends. J. Cell Biol..

[7] van Niel G, D'Angelo G, Raposo G (2018). Shedding light on the cell biology of extracellular vesicles. Nat. Rev. Mol. Cell Biol..

[8] Barile L, Vassalli G (2017). Exosomes: Therapy delivery tools and biomarkers of diseases. Pharmacol. Ther..

[9] Bobrie A, Colombo M, Krumeich S, Raposo G, Thery C (2012). Diverse subpopulations of vesicles secreted by different intracellular mechanisms are present in exosome preparations obtained by differential ultracentrifugation. J. Extracell Vesicles.

[10] Escola JM (1998). Selective enrichment of tetraspan proteins on the internal vesicles of multivesicular endosomes and on exosomes secreted by human B-lymphocytes. J. Biol. Chem..

[11] Belov L (2016). Extensive surface protein profiles of extracellular vesicles from cancer cells may provide diagnostic signatures from blood samples. J. Extracell Vesicles.

[12] Crescitelli R (2013). Distinct RNA profiles in subpopulations of extracellular vesicles: apoptotic bodies, microvesicles and exosomes. J. Extracell Vesicles.

[13] Kowal J (2016). Proteomic comparison defines novel markers to characterize heterogeneous populations of extracellular vesicle subtypes. Proc. Natl. Acad. Sci. U S A.

[14] Doyle LM, Wang MZ (2019). Overview of extracellular vesicles, their origin, composition, purpose, and methods for exosome isolation and analysis. Cells.

[15] Xu R, Greening DW, Zhu HJ, Takahashi N, Simpson RJ (2016). Extracellular vesicle isolation and characterization: toward clinical application. J. Clin. Invest..

[16] Corso G (2017). Reproducible and scalable purification of extracellular vesicles using combined bind-elute and size exclusion chromatography. Sci. Rep..

[17] Ross JF, Chaudhuri PK, Ratnam M (1994). Differential regulation of folate receptor isoforms in normal and malignant tissues in vivo and in established cell lines: Physiologic and clinical implications. Cancer.

[18] Luhrs CA, Slomiany BL (1989). A human membrane-associated folate binding-protein is anchored by a glycosyl-phosphatidylinositol tail. J. Biol. Chem..

[19] Sabharanjak S, Sharma P, Parton RG, Mayor S (2002). GPI-anchored proteins are delivered to recycling endosomes via a distinct cdc42-regulated, clathrin-independent pinocytic pathway. Dev. Cell.

[20] Grapp M (2013). Choroid plexus transcytosis and exosome shuttling deliver folate into brain parenchyma. Nat. Commun..

[21] Lotvall J (2014). Minimal experimental requirements for definition of extracellular vesicles and their functions: a position statement from the International Society for Extracellular Vesicles. J. Extracell Vesicles.

[22] Webber J, Clayton A (2013). How pure are your vesicles?. J. Extracell Vesicles.

[23] Raposo G (1996). B lymphocytes secrete antigen-presenting vesicles. J. Exp. Med..

[24] Thery C, Amigorena S, Raposo G, Clayton A (2006). Isolation and characterization of exosomes from cell culture supernatants and biological fluids. Curr. Protoc. Cell Biol..

[25] Issman L, Brenner B, Talmon Y, Aharon A (2013). Cryogenic transmission electron microscopy nanostructural study of shed microparticles. PLoS ONE.

[26] Linares R, Tan S, Gounou C, Arraud N, Brisson AR (2015). High-speed centrifugation induces aggregation of extracellular vesicles. J. Extracell Vesicles.

[27] Paolini L (2016). Residual matrix from different separation techniques impacts exosome biological activity. Sci. Rep..

[28] Momen-Heravi F (2012). Impact of biofluid viscosity on size and sedimentation efficiency of the isolated microvesicles. Front. Physiol..

[29] Van Deun J (2014). The impact of disparate isolation methods for extracellular vesicles on downstream RNA profiling. J. Extracell Vesicles.

[30] Garcia-Romero N (2019). Polyethylene glycol improves current methods for circulating extracellular vesicle-derived DNA isolation. J. Transl. Med..

[31] Patel GK (2019). Comparative analysis of exosome isolation methods using culture supernatant for optimum yield, purity and downstream applications. Sci. Rep..

[32] Gamez-Valero A (2016). Size-exclusion chromatography-based isolation minimally alters extracellular Vesicles' characteristics compared to precipitating agents. Sci. Rep..

[33] Alvarez ML, Khosroheidari M, Kanchi Ravi R, DiStefano JK (2012). Comparison of protein, microRNA, and mRNA yields using different methods of urinary exosome isolation for the discovery of kidney disease biomarkers. Kidney Int..

[34] Shu S (2020). Purity and yield of melanoma exosomes are dependent on isolation method. J. Extracell Vesicles.

[35] An M, Wu J, Zhu J, Lubman DM (2018). Comparison of an optimized ultracentrifugation method versus size-exclusion chromatography for isolation of exosomes from human serum. J. Proteome Res..

[36] Tauro BJ (2012). Comparison of ultracentrifugation, density gradient separation, and immunoaffinity capture methods for isolating human colon cancer cell line LIM1863-derived exosomes. Methods.

[37] Mathivanan S (2010). Proteomics analysis of A33 immunoaffinity-purified exosomes released from the human colon tumor cell line LIM1215 reveals a tissue-specific protein signature. Mol. Cell Proteom..

[38] Yang F, Liao X, Tian Y, Li G (2017). Exosome separation using microfluidic systems: size-based, immunoaffinity-based and dynamic methodologies. Biotechnol. J..

[39] Caradec J (2014). Reproducibility and efficiency of serum-derived exosome extraction methods. Clin. Biochem..

[40] Hessvik NP, Llorente A (2018). Current knowledge on exosome biogenesis and release. Cell. Mol. Life Sci..

[41] Smith ZJ (2015). Single exosome study reveals subpopulations distributed among cell lines with variability related to membrane content. J. Extracell Vesicles.

[42] Kuplennik N, Lang K, Steinfeld R, Sosnik A (2019). Folate receptor alpha-modified nanoparticles for targeting of the central nervous system. ACS Appl. Mater Interfaces.

