# Supplementary material for: High-grade extracellular vesicles preparation by combined size-exclusion and affinity chromatography
Source: Sci Rep. 2021 May 18;11:10550. doi: 10.1038/s41598-021-90022-y (PMC8131383; doi:10.1038/s41598-021-90022-y)

# High-grade extracellular vesicles preparation by combined size-exclusion and affinity chromatography

Cristina Bellotti[1], Kristina Lang[2], Nataliya Kuplennik[3], Alejandro Sosnik[3], Robert Steinfeld[1,2]

[1] *Department of Paediatric Neurology, University Children's Hospital Zurich, University of Zurich, Zurich, Switzerland*

[2] *Department of Child and Adolescent Health, University Medical Center Gottingen, Gottingen, Germany*

[3] *Laboratory of Pharmaceutical Nanomaterials Science, Department of Materials Science and Engineering, Technion-Israel Institute of Technology, 3200003 Haifa, Israel*

Corresponding author: Prof. Dr. Dr. med. Robert Steinfeld, robert.steinfeld@kispi.uzh.ch

ORCID IDs

Cristina Bellotti: https://orcid.org/0000-0002-3999-2807

Alejandro Sosnik: https://orcid.org/0000-0003-4704-4599

Robert Steinfeld: https://orcid.org/0000-0003-0956-6601

Supplementary data 1

Western Blot gels shown cropped in Figure 3 (Characterization of EVs obtained by FPLC in comparison to EVs isolated by the DC protocol).

DC: PTPS

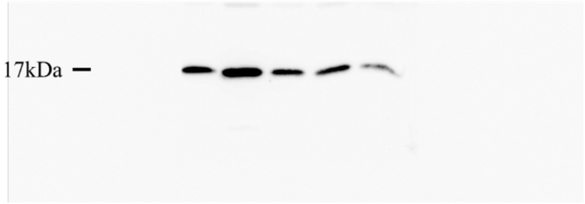


FPLC: PTPS

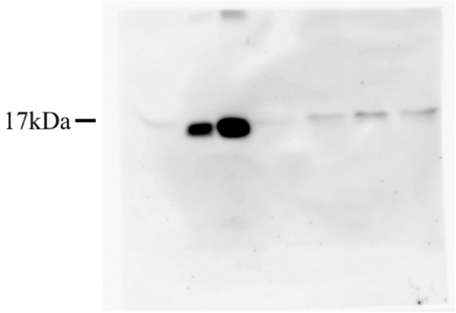


DC: TOM20

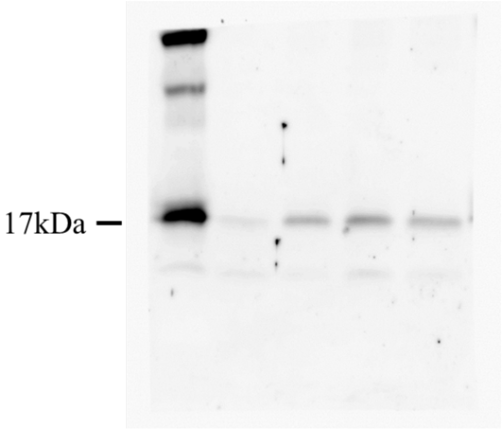


FPLC: TOM20

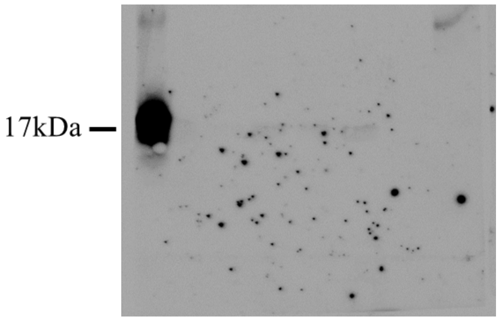


DC: Flotillin

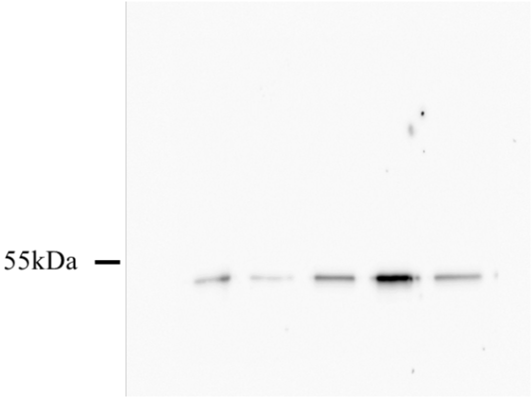


FPLC: Flotillin

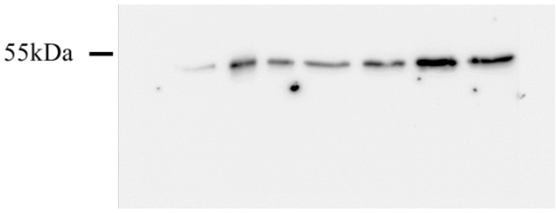

DC: Alix

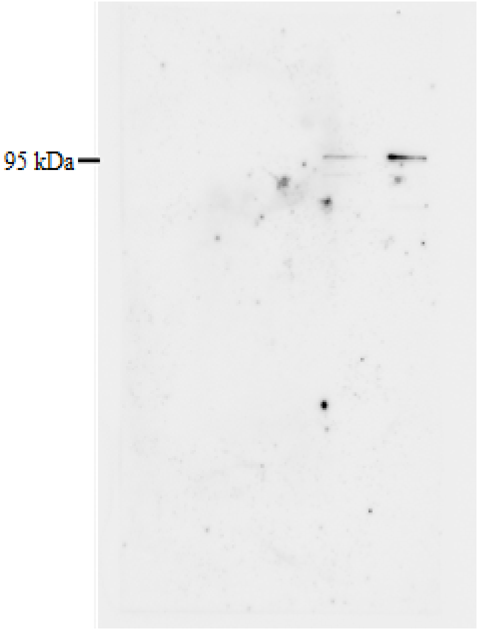


FPLC: Alix

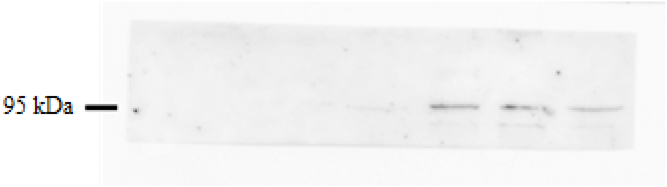


DC: FRα

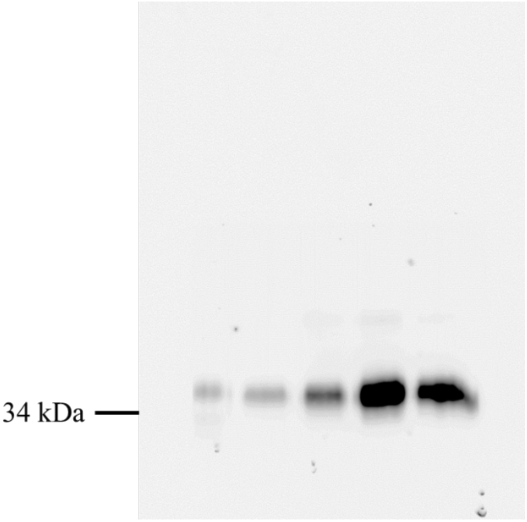


FPLC: FRα

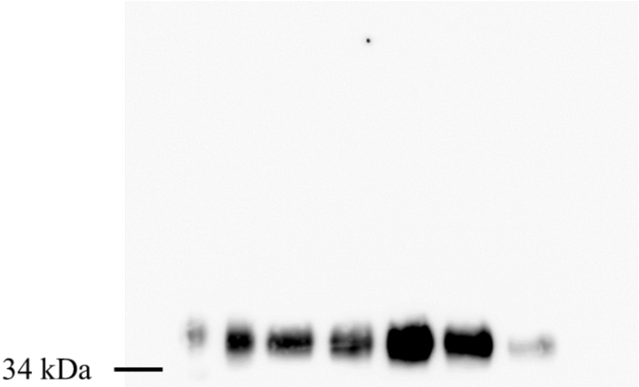

Supplementary data 2

Western Blot gels shown cropped in Figure 5 (Analysis of EV markers distribution after centrifugation on a sucrose gradient).

DC: Flotillin

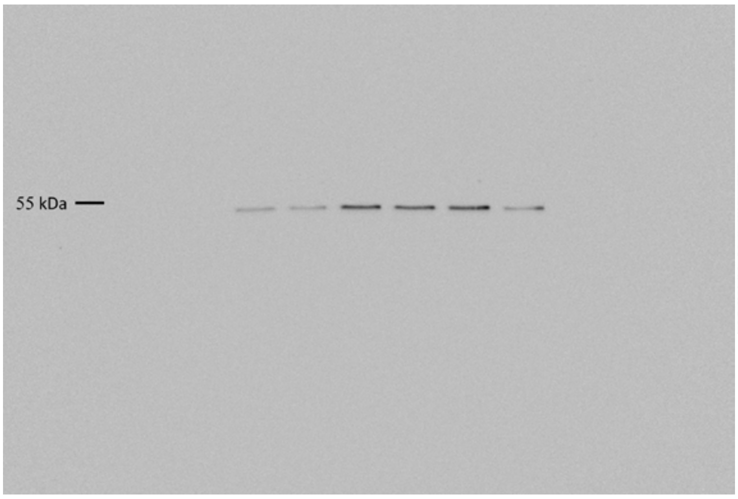


FPLC: Flotillin

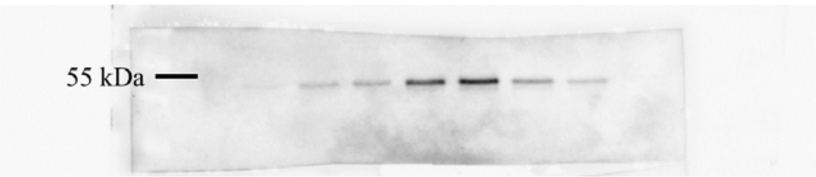


DC: FRα

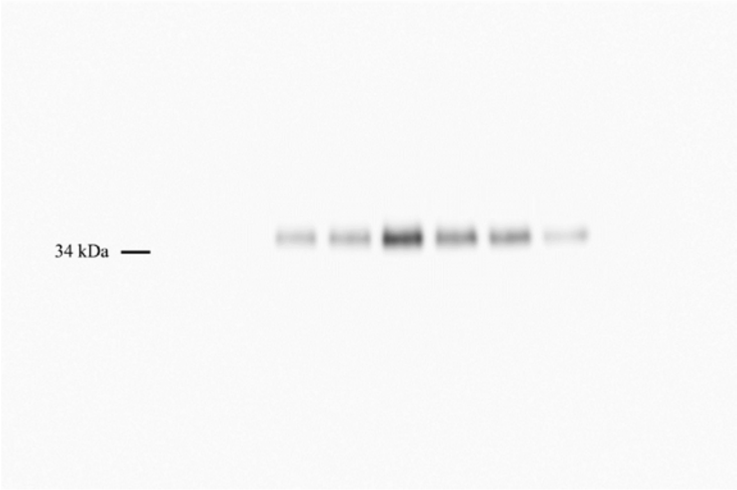


FPLC: FRα

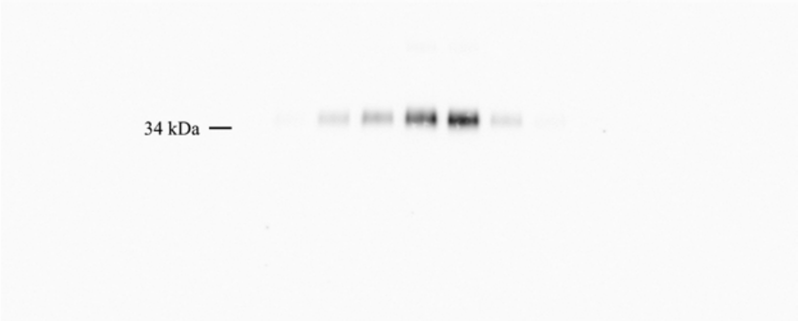

Supplement: Supplementary file 1 — Supplementary Information. [file 41598_2021_90022_MOESM1_ESM.pdf]
